# Deep Visible and Thermal Image Fusion for Enhanced Pedestrian Visibility

**DOI:** 10.3390/s19173727

**Published:** 2019-08-28

**Authors:** Ivana Shopovska, Ljubomir Jovanov, Wilfried Philips

**Affiliations:** TELIN-IPI, Ghent University - imec, St-Pietersnieuwstraat 41, B-9000 Gent, Belgium

**Keywords:** fusion, visible, infrared, ADAS, pedestrian detection, deep learning

## Abstract

Reliable vision in challenging illumination conditions is one of the crucial requirements of future autonomous automotive systems. In the last decade, thermal cameras have become more easily accessible to a larger number of researchers. This has resulted in numerous studies which confirmed the benefits of the thermal cameras in limited visibility conditions. In this paper, we propose a learning-based method for visible and thermal image fusion that focuses on generating fused images with high visual similarity to regular truecolor (red-green-blue or RGB) images, while introducing new informative details in pedestrian regions. The goal is to create natural, intuitive images that would be more informative than a regular RGB camera to a human driver in challenging visibility conditions. The main novelty of this paper is the idea to rely on two types of objective functions for optimization: a similarity metric between the RGB input and the fused output to achieve natural image appearance; and an auxiliary pedestrian detection error to help defining relevant features of the human appearance and blending them into the output. We train a convolutional neural network using image samples from variable conditions (day and night) so that the network learns the appearance of humans in the different modalities and creates more robust results applicable in realistic situations. Our experiments show that the visibility of pedestrians is noticeably improved especially in dark regions and at night. Compared to existing methods we can better learn context and define fusion rules that focus on the pedestrian appearance, while that is not guaranteed with methods that focus on low-level image quality metrics.

## 1. Introduction

Fully autonomous, artificial intelligence (AI)-based driving systems are currently one of the main promises for the future of smart transportation. A transition to fully autonomous vehicles, however, is likely to happen gradually, by increasing the degree of autonomy of current vehicles on the road. In the meantime, a human driver will remain present with important roles such as control takeover and situation monitoring, relying on advanced driver-assistance systems (ADAS).

Currently, driver-assistance technology is already being integrated into commercial and passenger vehicles, to assist a human driver and improve road safety [1]. Besides providing alert signalization and smart vehicle control, advanced driver-assistance systems provide the driver with the raw sensory information such as rear-view parking assistance cameras. Some car manufacturers have started integrating displays showing a feed from a thermal camera, to aid a human driver in challenging visibility conditions. In this paper, we will focus on fusion of visible and thermal images for improved visibility, targeting applications such as enhanced visual perception and future ADAS technologies.

Since the current commercially available sensors are limited in their spatial resolution and robustness for driving in various atmospheric conditions, diverse sensors are employed to capture more informative representation of the environment [1]. In applications such as autonomous vehicles and ADAS, multiple data sources are combined to improve situation awareness, obstacle recognition, night vision etc. Such sensors include visible and infrared cameras or radars.

A color camera performs well in good illumination conditions, providing rich color and detail information. However, poor light conditions compromise its effectiveness. At low light, the low signal-to-noise ratio (SNR) drastically degrades the image quality. To compensate for insufficient light the camera may increase the sensitivity of the sensor, the exposure duration, and/or the lens aperture. However, high sensor sensitivities amplify the noise, longer exposures increase the risk of motion blur and wide apertures narrow the depth of field resulting in unfocused regions. Moreover, while compensating for low light conditions, strong light from car headlights at night may cause glare. In addition to camera limitations, atmospheric conditions such as fog and mist diffuse the light and impair visibility.

Conveniently, long wavelength infrared (LWIR) or thermal cameras are not affected by the reflected visible light since they are sensitive to the heat emitted by objects. Thermal information is particularly helpful in distinguishing humans from their surroundings, as they tend to be warmer than the environment. However, when the scene and the objects in it are of relatively homogeneous temperature (for example on sunny days), important targets such as humans may become poorly distinguishable. Therefore, visible light and thermal cameras are considered complementary for vision systems operating in variable conditions.

In this paper, we propose a method for fusion of visible light- and thermal images, which produces a single blended image as an output, with the aim to preserve natural colors in the well-lit regions, and introduce artificial colors where objects are only visible to the thermal camera. Our goal is to increase the visibility of pedestrians by introducing information extracted from the thermal image, when thermal information is more reliable.

We train a single image fusion network that will be used as a stand-alone tool which produces enhanced images in difficult visibility conditions and thereby will assist a human driver. During training we propose to pair the fusion network to a second neural network pre-trained for pedestrian detection, which will not be re-trained and will only serve to define the error functions for the fusion network. The main novelty is that instead of relying on quality metrics, the auxiliary detection network will serve as a proxy for the cognitive processes by the human visual system. The goal is to present the resulting fused stream to a driver on a display, and not to an automatic decision system.

**Figure 1:**
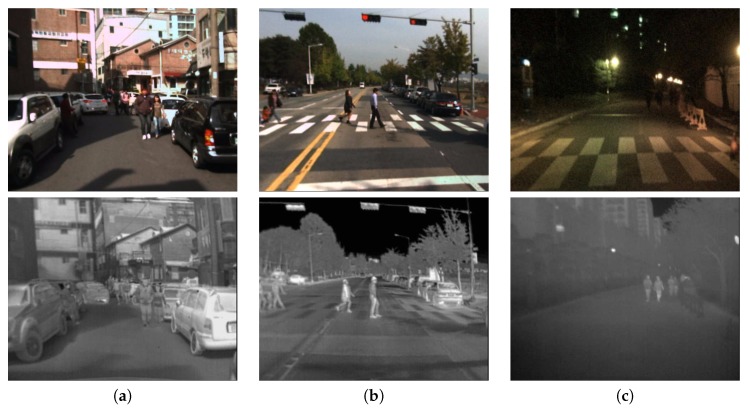
Examples of various light conditions and their influence on the visibility of people in visible light (first row)/thermal (second row) images: (**a**) Warm day with good visibility in bright regions and low contrast in the shadow in RGB; low contrast in thermal range. (**b**) Good pedestrian visibility in both visible light and thermal images. (**c**) Night scene with low visibility in RGB and pedestrians visible in thermal.

Presenting such enhanced video feed to a human driver on an in-car display allows the driver to rely on it in situations such as partial or complete lack of light when approaching a tunnel or poorly lit roads at night and make more informed decisions. Figure 1 presents an example of different visibility conditions that might be encountered while driving, and it illustrates easy and challenging examples for pedestrian visibility both for a visible light and a thermal camera.

The proposed method is conceptually different from the existing pixel-level image fusion techniques that use visible light- and thermal images in several aspects. In general, image fusion methods focus on maximizing the transfer of information from multiple input images into a single output, aiming to achieve good perceptual quality [2]. Two key components in image fusion are the activity level measurement and the actual fusion rules. In most methods these components are designed manually, based on empirical choices specific for a certain type of scene or application. However, the criteria to select the most adequate features and fusion rules may not necessarily be intuitive to humans, making it very difficult to manually come up with a design that optimally performs both activity level measurement and fusion. These problems compromise the effectiveness of the hand-crafted methods when applied to various scenes and conditions.

By relying on a learning-based framework and choosing a convolutional neural network (CNN) as a model, the proposed method performs automatic estimation of the optimal activity representation. CNNs are capable of modeling complex, nonlinear relationships and discovering new features of interest in the data, well suited for the application domain. Similarly, the actual fusion can be represented using more complex decision rules and learned jointly with the features. This provides CNNs with greater flexibility and performance advantage over conventional methods.

Additional difficulty in the domain of visible and infrared fusion is the definition of a reference image, or ground truth for comparison. Essentially, there exists no ground truth reference based on which the algorithms may be objectively compared. Moreover, different methods focus on different quality aspects and evaluate their performance using a set of no-reference objective image quality metrics. However, there is no single criterion that can guarantee superior performance for all types of scenes. Main application domains for most of the existing methods are surveillance and military applications, typically requiring accurate detail preservation and thermal intensity transfer in the final output.

The proposed method focuses on the quality aspect of visible and thermal fusion as well; however, our focus is directed towards maximizing the visibility of pedestrians in images of natural appearance for human drivers. To constrain the output to have natural appearance we optimize the fusion network with respect to the similarity between the output and the RGB input. The key difference compared to existing methods is that instead of relying only on low-level quality-based objective functions, we additionally use the detection performance of an auxiliary deep neural network for person detection as a training criterion. The auxiliary network is pre-trained for natural RGB images and fixed (non-trainable) during optimization. Our motivation for this is two-fold.

First, not all existing quality metrics are coinciding with the visual perception, and optimizing one criterion may decrease the quality with respect to another. For example, the problem of preserving edges without reducing the contrast is still not successfully solved for fusion of infrared and visible images [3]. This may pose a risk on the early visibility of the actual pedestrians while driving, despite having overall richness of details in the image.

Second, when optimizing low-level, information-theoretic, or statistical metrics such as transfer of details or average gradient, important relationships related to the appearance of pedestrians are ignored. Using a pedestrian detector as an objective allows propagating the focus on high-level properties such as shape, color/intensity, typical location etc. to the feature level, as well as in the fusion rules.

The main novelties of this paper listed below are related to the framework for training the fusion network:

1. The first novelty is the idea to optimize a pixel fusion method using a criterion for enhancing the visibility of objects of interest in the scene. The proposed method ensures that all relevant information for automated person detection is contained in the fused RGB output. We conjecture that this same information also enhances the visibility of pedestrians to humans.
2. We overcome the need for ground truth annotations at the pixel level by relying on an auxiliary detector network. We also overcome the need for ground truth detection annotations on multi-modal data by employing a regular detection network pre-trained on natural RGB images. This is important, since only a few annotated datasets exist for multi-modal pedestrian detection, without such annotations (e.g., semantic segment labels) available at the pixel level. While we focus on pedestrian detection, the approach can also be applied to enhance the visibility of any other object category, by replacing the auxiliary detector during training.
  The idea bears similarities to the concept of generative adversarial nets (GANs). The difference is that in the concept of GANs the generative network competes against a coupled discriminative network and both networks are optimized in alternation. In the proposed method we optimize only the generative (fusion) network, while the performance of the discriminator (detector) is a secondary goal. Another difference is that in the proposed method the discriminator is an object detector, while with GANs typically it provides a single decision on an image level, discriminating between natural or fake images.
3. As a pre-processing step we compute visual saliency maps for both RGB and thermal input images and add them as input of the fusion network. In the proposed method the saliency maps serve as general low-resolution guiding information for the detail activity in the images, emphasizing the regions of good visibility. Additionally, saliency helps in reducing the importance of the thermal information of the scene background at night when no details are present in the RGB input.
4. In the training process we aim to enforce the output to have high similarity to the RGB. Also, we want to retain the original image appearance in regions where pedestrians are clearly visible in the visible light domain, and only modify the result in regions of poor visibility. To achieve that, we modify the weight of the similarity-to-RGB loss function to have higher weight in pedestrian regions that are easy to detect in RGB.

Section 2 provides a review of existing work relevant for the problem of image fusion. In Section 3 we explain the idea behind the proposed fusion method into more detail, and in Section 4 we provide more technical details of the training setup. In Section 5 we explain the experimental setup and discuss the results. Finally, in Section 6 we summarize the paper and provide conclusions and directions for future development.

## 2. Related Work

Advanced driver-assistance systems (ADAS) are control systems integrated in vehicles to assist the driving task. ADAS have different principles of operation and levels of assistance for drivers [1]. Depending on the environment that is monitored, and the sensors used, ADAS can be categorized in different classes, the most recent of which are the surround-view, cooperative sensor networks with increased levels of driver support. These systems do not take actions fully autonomously, they only provide relevant information to drivers and assist them in performing critical actions. Combined with depth sensors ADAS can present the driver with additional data such as distance to objects and warnings for increased safety.

Current commercially available driver-assistance systems include Tesla Autopilot, Mobileye, and Openpilot that help in tasks such as collision avoidance, automatic lane centering, parking assistance etc. Similar to these solutions is the use of a thermal camera that is particularly beneficial for seeing in the dark. Systems such as Audi Night Vision Assistant, Mercedes-Benz Night View Assist, Toyota Night View and BMW Night Vision are some examples where thermal video is presented to the driver on a display in the car. However, relying only on the thermal vision at night is not sufficient due the lack of differentiating between colors which are significant for the road markings and traffic signs, and due to the low contrast between objects in the thermal range. In general, night vision assistance is actively being researched and the potential benefits of it are yet to be assessed [4].

The proposed method relies on front-looking cameras (referred to as exteroceptive sensors) and belongs to the type night-vision enhancement systems, where information is presented on a screen for a visual support. Our current focus is on the support by enhanced visualization of the visible scene by fusing visible and thermal images, and not for processing by automatic, internal algorithms. In future development, the proposed framework could also be incorporated in automatic recognition by intelligent driving systems.

Fusion of visible and thermal images to enhance the visibility of objects of interest has received little attention in the literature. Predominantly, existing methods focus on enhancing the quality and the amount of information in the output image, optimizing various global quality metrics. The core building blocks are effective information extraction and fusion rules with the goal to achieve improved visibility without introducing any artifacts.

Paper [2] provides a comprehensive overview of existing techniques for visible and infrared image fusion. The majority of the existing approaches [5,6,7,8,9,10,11,12,13,14] are estimating fusion weights based on low-level image features, used for mapping the pixel intensities of the input images to the output. In early methods these weights were estimated based on empirical choices, without any criteria to evaluate the features.

More recent approaches for information extraction and definition of weights are mainly learning-based, where optimization is performed with respect to desired perceptual quality measures [15,16,17,18,19,20,21]. Our goal on the other hand is fusion of the visible- and thermal band images, optimized with respect to the salient features of the pedestrians.

Vast majority of the prior research efforts were focused on surveillance systems, while in recent years automotive applications are becoming dominant in the literature. Several application-oriented methods [22,23,24,25,26] perform fusion of visible light and thermal images for specific automotive applications such as pedestrian detection or traffic monitoring. However, they rely on a limited number of image transformations for decomposition and on hand-crafted algorithms for fusion. Despite being designed to facilitate object detection and image analysis, the performance of the existing analysis algorithms is only evaluated during the testing phase and is not directly coupled with the optimization of the fusion algorithms. As a result, the optimal algorithm performances may not be realized.

In the literature, saliency estimation is a common building block introduced into the fusion process to improve the visual quality of the fused images [13,20,21]. Saliency provides information about the features that attract human visual attention, often limited to an object in the foreground. However, a very precise and accurate saliency estimation remains an open challenge. Using the saliency of the input images for image fusion is an additional challenge in the image fusion literature.

In the proposed method, due to its simplicity in calculations, saliency is estimated using the method proposed by Hou et al. [27] and used as an additional input into the neural network. We compute saliency for both visible light and thermal inputs and obtain low-resolution maps that indicate regions of higher visual importance. The neural network can thus learn how to use the saliency maps to combine the inputs more efficiently and to increase the visibility of important objects.

Apart from direct low-level image fusion, some methods presented in the literature have been exploring fusion at a higher semantic level to improve object detection. The existing high-level fusion methods typically define fusion as a classification problem with multi-modal input [28,29]. In recent works, the general approach resulted in training discriminative deep CNNs for pedestrian detection in visible and infrared inputs [30,31,32,33,34,35]. Fusion is performed in hidden layers, typically showing best performance when applied at the feature level. However, the multi-modal detectors are still not robust and accurate enough to fully rely on them while driving. Also, these detectors do not provide an intermediate output comprehensible to a human driver that could be used in driver-assistance systems.

In contrast to existing methods, the proposed method focuses on improving the visibility of objects of interest, specifically pedestrians. In absence of ground-truth fused images or segmentation with semantic labels, our method relies on automatic pedestrian detection for extracting relevant visual information. Still, improved visualization to assist a human driver remains our main goal.

Most existing pedestrian detectors are trained using regular RGB images, while in the proposed method they will be applied to fused images. This means that the auxiliary detector should have some robustness with respect to the appearance of pedestrians. The motivation for this approach is the broad availability of annotated RGB data, compared to the limited availability of thermal datasets required to robustly train a detector.

Detectors that rely on contextual information in addition to pedestrian appearance are most suitable in this respect. Among the papers in the literature [36,37] CNN-based detectors achieve the best performance. Recent focus on fine-grained image classification resulted in several works proposing attention-based networks for spatial and part-based object recognition [38]. In this paper, we will rely on Faster R-CNN [39] trained for regular RGB images as our auxiliary detector. Faster R-CNN is a multiple-category object detector that is composed of three parts: feature extraction, region proposal, and classification. Its design that combines several individual loss functions makes the training tractable and the network more easily adaptable.

In [28], a labeled multi-spectral dataset for automotive applications was established, known as the KAIST multi-spectral pedestrian dataset. The dataset contains driving sequences recorded both at day and at night, accompanied by pedestrian bounding-box annotations. This paper has set the baseline for comparison of detection algorithms employing visible light and thermal image pairs. In this paper, we will use the multi-modal image pairs of the KAIST dataset as inputs to the fusion neural network, while the provided pedestrian annotations will be used to evaluate the detector in the training process.

Among the deep learning techniques, discriminative models have achieved significant success in various classification tasks. Generative models, on the other hand, have had more challenges with model estimation, especially when ground truth information is not available. An important contribution for generative model training was proposed in [40]. In general, GANs are employed to learn a mapping that transforms a sample from a source distribution to a new sample from a target distribution. A recent paper [41] has addressed the issue of image fusion from a GAN perspective, proposing a method called FusionGAN. In this method the generator aims to produce a fused image with infrared intensities combined with visible gradients, while the discriminator aims to force the fused image to contain more texture transferred from the visible light. This method still focuses on low-level quality features such as texture.

In terms of improving image quality, in [42] a no-reference quality estimation problem is also tackled with a generative adversarial approach. The role of the generator is to learn to generate a high-quality reference output given a distorted input. The discriminator is trained to estimate the quality score of the degraded image based on the discrepancy with the generated reference image. This paper is an example where the role of the generator is to help improve the performance of the discriminator.

Our CNN-based image generator has a similar goal as the earlier learning-based techniques for improved visibility, while the model estimation process is closer to the GAN concepts. In the proposed method, the fusion network (generative) is trained to produce three-channel RGB images with high resemblance to the original RGB inputs to preserve natural appearance and intuitive results. At the same time, it is trained to modify the original RGB values in the pedestrian regions, so that the visibility of pedestrians is increased. This is achieved by coupling it to a pedestrian detector network (discriminative) as a measure of the degree of visibility of the pedestrians in the fused image. While keeping the detector fixed, the error gradients are propagated back to the fusion network to optimize the parameters of the latter.

## 3. Learning-Based Image Fusion

In the following sections we will explain into more details the proposed architecture of the image fusion network, as well as the novel framework for training it to combine RGB and thermal inputs into a new, more informative fused output.

### 3.1. Proposed Generative Fusion Network

Deep CNNs have proven highly powerful in encoding spatial and spectral relationships and learning complex data associations between the input and the target output. Moreover, they do not require explicit definition of features, rather, they are able to learn the parameters of the convolution operations that define the features.

Recently, the residual learning approach [43] has shown important advantages in the training of deep neural networks. Residual learning is realized by including connections in the neural network that copy and add the input from one layer to the output of another layer later in the cascade. That way the intermediate layers learn to model only the *residual* between the input and the output of the residual block. This architecture has proven to facilitate the training and reduce the error, since it allows modeling more complex relationships in the data without having to model the input itself through the layers.

**Figure 2:**
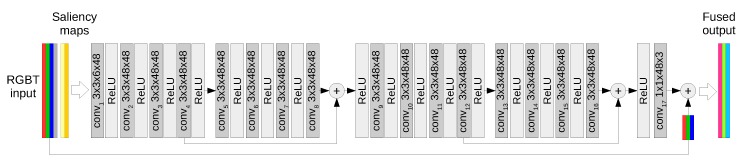
The architecture of the fusion network is an array of high-resolution convolution layers with residual blocks. The input is a concatenation of the input RGB+Thermal channels and saliency maps computed on the luminance of the RGB and on the thermal intensity. The fused result is obtained by adding the residual output of the network to the original RGB input.

The proposed fusion function is modeled using a generative convolutional neural network, depicted in Figure 2. It is a feed forward, high-resolution network consisting of convolutions followed by rectified linear units (ReLUs) that allow modeling nonlinear functions.

Motivated by the success of residual learning and its compatibility with our goal, we incorporate residual learning by forming residual blocks in the cascade of layers. Additionally, we include a copy-and-add operation which connects the original RGB input and the fusion output. This way the network inclines towards focusing on the content that contains additional information to the original RGB image to increase the visibility in regions with pedestrians, leaving all other areas unaffected.

As shown in Figure 2, the input to the network is composed of an RGB image and the corresponding, aligned LWIR image. In addition, we propose to enrich the input with saliency maps computed from the RGB and thermal images, resulting in a 6-channel input $I^{M\times N\times 6}$. The convolution filters of the network are of size 3×3×48 which creates feature maps of 48 dimensions, which is factor 8 increase compared to the input. The final layer of the network consists of three 1 × 1 convolution filters that map the multi-dimensional features into a three-channel output $I_{F}^{M\times N\times 3}$.

Computing saliency in the visible light images facilitates distinction of regions with good visibility and contrast. As a pre-processing step we apply the saliency detection method based on the so-called image signature, proposed by Hou et al [27]. This method is simple to calculate, while producing results closer to human attention model compared to previous methods.

In [27], the image is considered to be a mix of a foreground signal embedded in a background signal. The basic assumption is that the foreground signal is sparsely supported in the spatial basis, and the background is sparsely supported in the basis of the Discrete Cosine Transform (DCT). This method relies on the image signature, which is defined as the sign function of the DCT of the image. The saliency map is obtained by smoothing the result of the inverse DCT of the image signature. The authors have experimentally proven that the estimated saliency predicts well the locations capturing the human visual attention.

In the proposed method, the saliency is calculated on a low resolution (in our method experimentally set to 52×64). The low-resolution saliency maps are then resized to match the input image resolution before including them as inputs to the neural network. They serve as rough guiding information representing the foreground of the scene which is relevant for our application. For example, in thermal images saliency helps to focus on objects of strong temperature contrast such as humans in the scene and to suppress the influence of large warm background regions such as roads and buildings. At nighttime, the saliency of the visible light images helps to deprioritize dark regions.

Our aim is to train the network under various visibility conditions to make it robust and applicable to realistic driving situations. Training it in variable conditions helps the network to learn how to effectively extract information from each modality. For example, in good visibility conditions the output should be similar to the RGB image since it contains more rich details and colors. At night typically the only available information for the pedestrians is present in the thermal image, and therefore this thermal information will need to be added to the background scene.

### 3.2. Proposed Detection-Guided Learning

Our main goal is to train the network to identify which structures, shapes, or intensities in each input are relevant for improved object visibility. However, there exists no ground-truth representing the ideal fusion result in supervise the learning. On the other hand, we want to refrain from fully relying on image quality criteria.

The main novelty of the proposed method is that the objective function is proportional to a measure of detection success. To incorporate a classification performance measure in the optimization framework, we propose to apply an auxiliary detector with differentiable loss function, whose error gradients can be computed and propagated back to the fusion network. The auxiliary detection network is required only during the training of the fusion network. In visualization applications, the fusion network can be used as a stand-alone tool to assist a human driver who will observe the enhanced scene.

**Figure 3:**
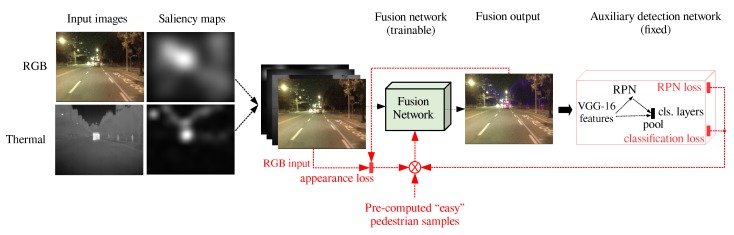
Diagram of the detection-guided learning of the fusion function. The red color represents the operations performed during training related to estimation of the losses. Both appearance and auxiliary detection losses are combined to train the fusion network. Please note that the auxiliary classification network is only used during training.

The design of the proposed framework for training is presented in Figure 3. As the figure shows, the output of the fusion network is connected to the auxiliary pedestrian detector, so that the 3-channel fusion output is input for the detector. We adopted the Faster R-CNN model [39], a deep neural network that is trained to locate and recognize different classes of objects, including pedestrians, in regular RGB images. Since our goal is to generate images with natural appearance, the Faster R-CNN model is compatible with our fused outputs. Also, Faster R-CNN is compatible with our formulation of guide information based on natural person appearance.

As explained before, the output of the fusion network should be a color image with natural appearance, similar to the RGB input. At the same time, the network should ensure that pedestrians are clearly visible, either by retaining the pedestrian regions similar to RGB in good visibility conditions, or by introducing false colors to create contrast at low visibility, based on the thermal image.

We define separate loss functions for the overall image appearance and the pedestrian regions. The different losses are computed at different locations of the joined training architecture, and they are all minimized simultaneously. The appearance loss is computed at the last layer of the fusion network, while the loss related to pedestrian detection is computed at the output of the detection network. Effectively, during backpropagation, the gradients of all losses are summed at the last layer of the fusion network and propagated back to adapt the parameters of the fusion network. The parameters of the auxiliary detector remain fixed.

The loss related to the overall appearance of the fused output image aims to enforce that the fused image resembles the original RGB input. We define the appearance loss $L_{a}$ as the mean squared error between the color values of the fused output image $I_{F}$ and the RGB input image *I*, averaged over all image pixels x=[1..N], and all three channels *c*:

(1) $$L_{a}=\frac{1}{3N}\sum_{c\in \left\{R,G,B\right\}}\sum_{x=1}^{N}{\left[I_{F}^{c}\left(x\right)-I^{c}\left(x\right)\right]}^{2}$$

As a result, of the residual learning approach, minimizing $L_{a}$ alone will essentially force the fusion network to directly forward the RGB input to the output, completely ignoring the thermal image by setting the network parameters to zero and posing a risk for problems such as vanishing gradients. Therefore, we minimize this loss simultaneously with the loss of the auxiliary detection network as explained below.

Since multiple loss functions are combined to train the network, all will lead the training towards a different solution. However, at the same time we want to ensure that the pedestrians of good visibility will remain mostly unaffected by the influence of the thermal input. To achieve that, we pre-process the dataset using the auxiliary pedestrian detector and identify samples that should not be modified by fusion due to their good visibility in RGB. We define easy samples that are correctly detected with a score above an experimentally determined threshold of 0.75. During training, we increase the weight of the appearance error term 10 times in these regions, to force the output to retain the original RGB values.

Faster R-CNN [39] is a merge of a region proposal network (RPN) and a classifier network based on its predecessor Fast R-CNN network [44]. The RPN and the classification sub-network share the output of a block of convolutional layers (based on the VGG-16 model [45]) that produces low-resolution, multi-dimensional feature maps.

Using the feature maps, the region proposal network generates an initial pool of regularly spaced, rectangular regions of different sizes and aspect ratios denoted as anchors. The RPN classifies the anchors into two categories, object (of any class) or background, and proposes adapted bounding-box dimensions. The regions classified as positive (objects) are denoted as proposals. The output of the RPN is a set of 4-element vectors containing bounding-box coordinates and sizes of the positive samples, and a set of corresponding scalars that represent the decision scores.

The classification sub-network of Faster R-CNN aggregates the shared features on the locations of the proposals and classifies them into multiple categories. This sub-network also outputs 4 coordinates and a classification score for each positively classified proposal, similarly to the RPN.

In [39], to train Faster R-CNN two equivalent multi-task loss functions are minimized: $L_{rpn}$ for the RPN and $L_{frcnn}$ for the classifier. In the proposed approach we will not train Faster R-CNN, but only rely on its losses simultaneously, presented in Equations (2) and (3).

In both equations the multi-task loss is a weighted average of two losses: log loss for the class scores $L_{cls}$, and Huber loss for the regressed bounding-box coordinates $L_{reg}$:

(2) $$L_{rpn}\left(p^{A},t^{A}\right)=\frac{1}{N_{cls}^{A}}\sum_{i}L_{cls}\left(p_{i}^{A},p_{i}^{*}\right)+\lambda_{1}\frac{1}{N_{reg}^{A}}\sum_{i}p_{i}^{*}L_{reg}\left(t_{i}^{A},t_{i}^{*}\right)$$

(3) $$L_{fastrcnn}\left(p^{P},t^{P}\right)=\frac{1}{N_{cls}^{P}}\sum_{j}L_{cls}\left(p_{j}^{P},p_{j}^{*}\right)+\lambda_{2}\frac{1}{N_{reg}^{P}}\sum_{j}\left[p_{j}^{*}\geq 1\right]L_{reg}\left(t_{j}^{P},t_{j}^{*}\right)$$

In Equation (2), the arguments $p^{A}=\left\{p_{i}^{A}\right\}$ and $t^{A}=\left\{t_{i}^{A}\right\}$ are the predicted class probabilities and the corresponding bounding-box coordinates of the positive anchors *i*, respectively. Since the RPN is a binary classifier, $p_{i}^{*}$ is a ground-truth scalar label with value 0 or 1 depending whether the predicted region was associated with a negative or a positive ground-truth bounding box with coordinates $t_{i}^{*}$.

Similarly, in the classifier loss Equation (3), $p^{P}=\left\{p_{j}^{P}\right\}$ and $t^{P}=\left\{t_{j}^{P}\right\}$, are the class probabilities and bounding-box coordinates, evaluated for positive *proposals* *j* with coordinates $\left\{t_{j}^{*}\right\}$. In this case, the multi-class ground-truth labels $\left\{p_{j}^{*}\right\}$ are *vectors* with binary elements, with value 1 for the true object category and 0 for all others. The coordinates of the ground-truth bounding boxes corresponding to the positive proposals are $t_{i}^{*}$. The term $\left[p_{j}^{*}\geq 1\right]$ is an indicator function and equals to 1 if the sample is positive for a given category and 0 if negative.

In Equations (2) and (3), the normalization constants are determined following the approach of the paper that proposed Faster R-CNN [39]. The RPN terms are normalized by the mini-batch size $N_{cls}^{A}$ and number of anchor locations $N_{reg}^{A}$. In the classifier loss terms, $N_{cls}^{P}$ is equal to the number of selected training proposals and $N_{reg}^{P}$ to the total number of generated proposals. The coefficients $\lambda_{1}$ and $\lambda_{2}$ determine the weight of each term in the overall loss. The authors of Faster R-CNN found that modifying these coefficients did not make significant difference in the results, so we fixed them to 1. Since in this paper we focus only on pedestrians, we ignore the terms of the classification loss unrelated to the pedestrian category.

The two multi-task loss functions in Faster R-CNN have different effects on the fusion output. Minimizing the region proposal error leads towards a solution with higher detail activity and larger set of proposals, but not exclusively related to the appearance of humans. Minimizing the classification error on the other hand leads towards a solution that makes the proposed pedestrians more distinguishable from other object categories. We have trained the network using the weighted mean of both errors, with an experimentally determined trade-off of 1:10, giving higher significance to the classification errors.

**Figure 4:**
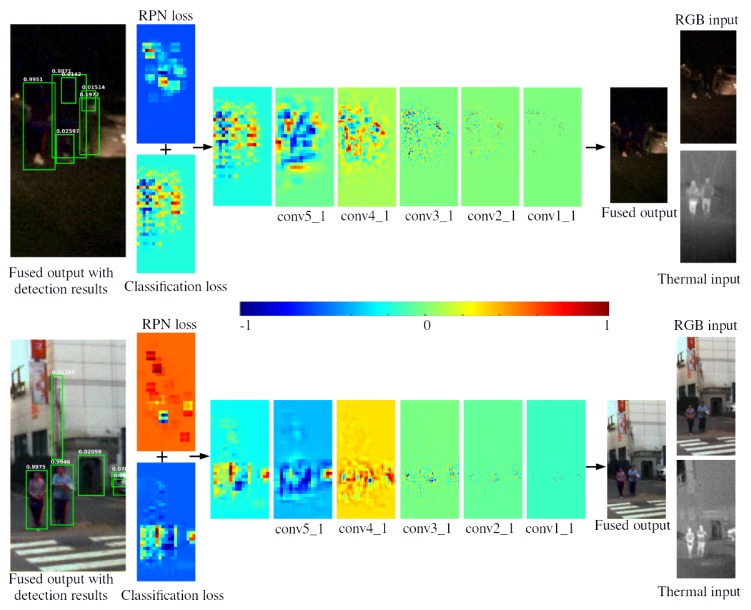
Normalized examples of the error gradients for the fused outputs calculated from the detection loss. The figure shows the propagation of the gradients from the region proposal and classification sub-networks through the VGG-16 feature layers, towards the trainable fusion network. The values shown here are normalized in the range [-1,1] for each map separately for visualization purposes.

During backpropagation, the error gradients originating from the RPN and classification parts are summed at the first (in reverse order) common convolution layer from the VGG-16 graph. An example of the gradient magnitudes from the region proposal and classification modules is shown in Figure 4. The loss is back-propagated through the VGG-16 feature layers towards the fusion network, from coarse to fine resolution. For illustration, all feature channels in the corresponding convolution stage were summed into single-channel maps, and the low-resolution maps were up-sampled. We show the relative values in a normalized range [-1,1] for each map independently.

In the examples in Figure 4, the image on the left shows a crop from a fusion result together with the result of the detection. It can be noticed that the gradients are the strongest in the regions where pedestrians are located. The RPN loss is different from the classification loss since it is designed to classify objects belonging to multiple categories, and operates on lower resolution. The last gradient map resulting from conv1_1 is the loss gradient directly fed to the fusion layers. Regarding the design of loss functions, the main novelty of our approach is that this information is being exploited by the fusion network, acting as a gradient of the fusion quality loss.

## 4. Training Setup

Since there is no analytic solution to this problem, the minimum of the loss can be found by iterative methods such as gradient-based methods. Minimizing the error consists of finding the point in the multi-dimensional solution space where the loss function reaches a minimum. The location of the minimum is defined as a point in the solution space where the derivative of the loss with respect to the network parameters is zero. Gradient methods are iterative optimization algorithms that estimate the search direction towards the global minimum by relying on partial derivatives of the error function. A popular technique used in the deep learning literature that overcomes several issues typical for early gradient-based techniques is the Adaptive Moment Estimation (ADAM) [46]. ADAM computes adaptive learning rates for each parameter, and keeps track of exponentially decaying average of past gradient moments. We have found that ADAM compared favorably to other optimization algorithms with respect to our application, so we adopted it as a training method of the fusion network.

The Faster R-CNN model that we use as auxiliary loss was pre-trained using the Microsoft COCO (Common Objects in Context) dataset [47], which is a large collection of color images that contain objects belonging to numerous different categories. However, for the current application we will use multi-modal inputs and single-category (pedestrian) labels to estimate all losses necessary for training the fusion network. The auxiliary detector will not be re-trained, and will be applied to the *fused* RGB outputs.

The KAIST Multi-spectral Pedestrian Detection Benchmark [28] provides a collection of video sequences with corresponding ground truth pedestrian annotations. We used both daytime and nighttime images from the dataset to learn a general model applicable to variable conditions regardless of time of the day. The order of the images was randomized to speed up convergence, since the training set contains multiple consecutive images from similar scenes. Among the annotations, apart from the label “person” and “cyclist” there are regions labeled as: “person?” and “people”. These are samples for which the human annotators were not certain of the presence or absence of a person, or when a group of indistinguishable people was labeled. To avoid ambiguity during training, we modified the default proposal sampling method and did not sample these regions during computation of the losses.

Relying on the detection criteria from Faster R-CNN imposes difficulties in the model estimation due to several reasons. First, the problem is ill-posed as multiple solutions exist. In our specific case, we aim to achieve a natural image appearance and at the same time satisfy optimal detection criteria. Therefore, training simultaneously with the appearance loss will lead the solution towards a model that generates results similar to regular RGB images and at the same time optimizes the detection losses.

Another aspect that we have considered is that the loss functions of Faster R-CNN penalize both the false positive and the false negative outcomes. Since the proposed method is mostly concerned with the information that needs to be extracted to increase the visibility of pedestrians in challenging conditions, we generally focus on improving the recall rate of the classifier. However, reducing false negatives is also important so that the network does not introduce artificial, human-like structures in non-person locations. We have experimentally chosen a trade-off for training. In the first 2 epochs we perform training based on the error computed only from the positive samples (i.e., the errors of true pedestrians), later increasing the number to a limited set of around 5 ROIs (including positive and negative samples), gradually ending with approximately 20 ROIs per image.

From the full KAIST training dataset, 14,300 images which contain at least one person were selected as a training set. The fusion network was trained image per image, iterating 10 epochs through the full training set. The initial learning rate was 0.001, later decreased to 0.0001 for the final 3 epochs.

## 5. Experiments and Results

The main criteria for the evaluation are the visual properties of the fused images resulting from the learned fusion function. As previously explained, with the proposed training framework we rely on the auxiliary pedestrian detector to substitute a high-level cognitive perceptual metric correlated with the human vision. Since we do not have such a metric, the fusion network was trained to optimize features relevant for the detector and improve the performance of the detector. However, our main goal is improved visibility for interpretation by humans, and therefore we apply some post-processing operations to increase the contrast of the details generated by the network.

We perform the proposed post-processing on the residual information generated by the fusion network. First, to suppress noise we perform guided filtering of the residual image RES using itself for self-guidance and obtain $RES_{GF}$. This has the effect of edge-preserving smoothing of the image. The guided filtering is followed by multiplication by 3 and a gamma function to the residual (γ=1.5) to increase the brightness and contrast of the residual information that is added to the RGB image to create the fused output:

(4) $$FUSED=RGB+{\left(3\times RES_{GF}\right)}^{\gamma }$$

**Figure 5:**
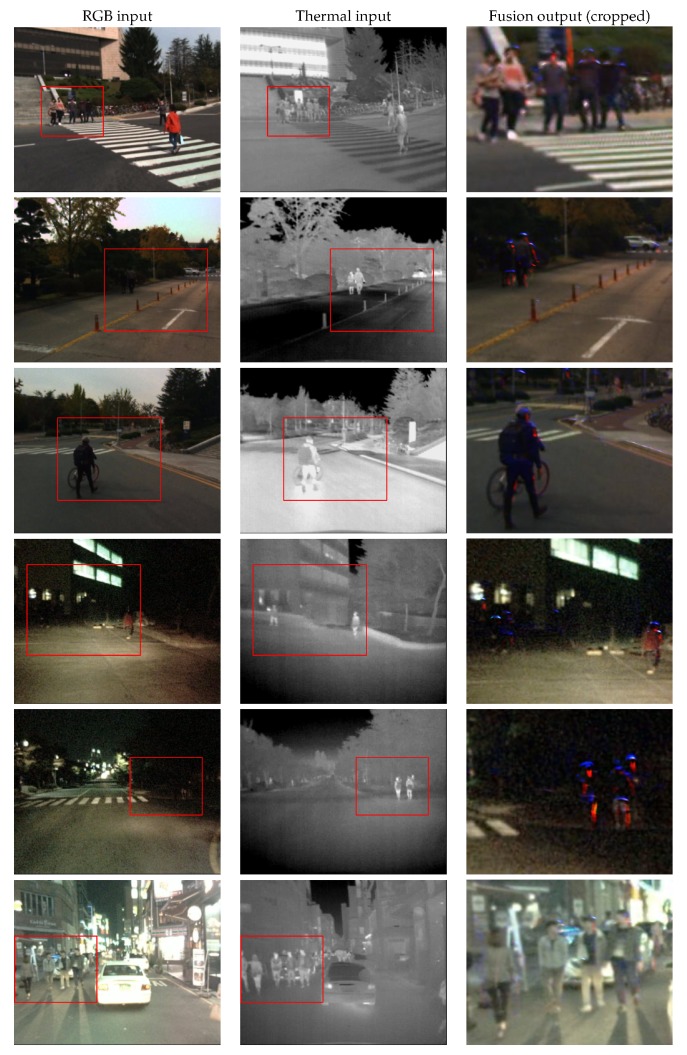
Daytime and nighttime examples of the inputs from the KAIST dataset and the corresponding fused outputs obtained with the proposed method.

Figure 5 presents some examples of daytime and nighttime images obtained by fusion of visible light and thermal images with the proposed approach. The post-processing operations and their parameters mainly depend on the cameras used to capture the input images, the levels of noise, and the compression artifacts. For the presented examples we have empirically determined the parameters of Equation (4). In the first and second column we show the inputs to the fusion network, with the red rectangles marking the more important regions of the images which are then shown cropped and enlarged in the samples of the fusion results in the third column.

The post-processing step amplifies the intensity of the details added to the RGB image, and creates a higher contrast with the background. It is evident that the fusion network has learned to make pedestrians visible in nighttime images by applying false colors, while at daytime in images with good visibility the pedestrians are less affected by false colors and more similar to the visible light image. The edges of the pedestrian silhouettes are accentuated by contrasting colors. This kind of output can be displayed on an in-car assistance system, so that a human driver can rely on it in challenging conditions for early risk prevention.

**Figure 6:**
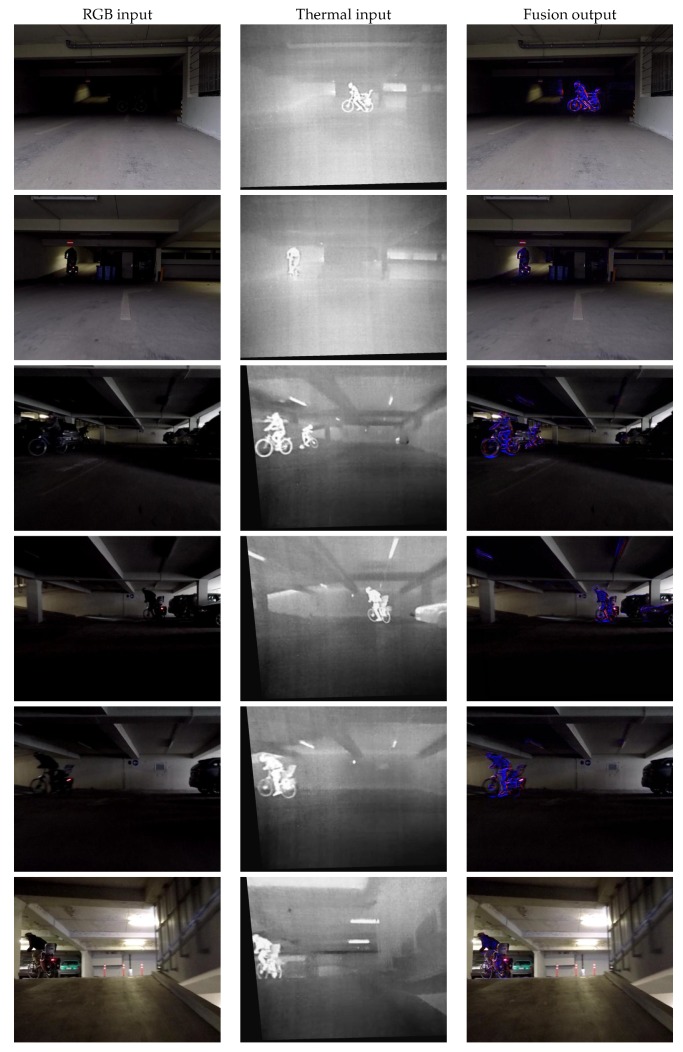
Cropped frame samples from our own sequence recorded in a parking lot using a pair of GoPro and FLIR cameras.

Figure 6 presents samples obtained in an original data collection session, from a moving platform. Here we show crops of the original frames for closer illustration of the important results. The inputs are presented in the first two columns, and the fusion results are presented in the third column.

The RGB images were recorded with a GoPro Hero 6 camera (GoPro, Inc., San Mateo, CA, USA), while the thermal using a FLIR ThermiCam BPL 390 (FLIR Systems, Wilsonville, OR, USA), mounted next to each other horizontally. The video sequences of both modalities were captured with a frame rate of 30 frames per second. They were then synchronized and calibrated by applying a pre-computed, global geometric transformation to match the viewpoints as closely as possible.

With this experiment we aimed to visually evaluate the results in a more realistic scenario, and on completely different data than the training dataset. This collection has not been annotated with object labels; however, it is of higher quality than KAIST in terms of noise and image resolution. Moreover, we present a situation in which both the visible and thermal information are crucial for safe driving at the same time.

While the RGB contains more rich details of the environment, the thermal frames capture well the humans in the dark scene. From the results presented in the third column in both Figure 5 and Figure 6 it can be noticed that in dark surroundings the human objects were strongly accentuated by our fusion network, while the original appearance of the surrounding area remains visible. In cases of good contrast of the person in the color image, the thermal influence is less strong (Figure 5 rows 1 and 6 and Figure 6 rows 2 and 6) and the original visible light appearance is retained.

In terms of quantitative evaluation, using standard quality metrics, we have compared the results of our algorithm to ten recent fusion methods with publicly available codes: [5,6,7,9,10,11,12,13,14]. These methods were designed for direct, pixel-based image fusion for improving the overall quality of the output. We have used each implementation to fuse and test the images of the KAIST test set and calculated the average of each metric.

Most of the reference methods to which we compare the proposed work were designed to fuse grayscale visible light and thermal images. Since our experiments involve three-color visible light inputs plus a single-channel thermal image and three-color fused outputs, we first convert the RGB inputs to a grayscale representation. We transform the input RGB colors to the CIE Lab color space and use only the luminance channel to represent the grayscale version of the visible light images. The grayscale images are used as the inputs for the compared methods from literature. Since our method uses three-color visible light images, for consistent comparison we convert the three-channel output of our network to a grayscale representation and calculate the quality metrics.

In the image fusion literature, there is no standard quality metric used for comparison between methods. Therefore, all methods are typically evaluated using a set of multiple metrics. We have evaluated our fusion method using several popular objective quality metrics [48]. The metrics include: Mutual Information (MI) [49], gradient information preservation ($Q^{AB/F}$), Information Entropy (IE) [50,51], Average Gradient (AG) and Spatial Frequency (SF). Information-theoretic metrics such as AG, IE and SF essentially evaluate the amount of details and variability in the fused image, reasoning that images with higher levels of detail have higher perceptual quality. On the other hand, metrics such as MI and $Q^{AB/F}$ measure the amount of information that is being transferred from each input into the fusion output (information being defined by intensities and gradients, respectively). The higher the amount of transferred information, the higher the quality of the result.

The results are presented in Table 1, with the bottom row presenting the results of the proposed method. Higher numbers correspond to better quality, and the best performances are indicated with bold numbers. The last two columns are part of the next experiment which will be explained later.

Due to differences with the existing methods in the definition of the fusion goal, the estimated quality of the proposed method varies in these metrics. For example, our method achieves very high MI score due a high similarity between the fused images and the input RGB images. On the other hand, preservation of all gradients from the thermal images was not our primary goal and therefore, mainly the pedestrian regions are enriched with details. Nevertheless, the proposed method still ranks relatively high in all columns, indicating that it does not degrade the image quality and transfers information from both input modalities.

In the absence of a reliable human perception model, as additional experiment we have evaluated the fusion results with respect to automatic person detection. We assume that enhancing the information relevant for automatic detection at the same time enhances the visibility of pedestrians to humans observing the image. In line with this assumption, in the last two columns of Table 1 we compare detection performance achieved on the fused images of the KAIST dataset, fused using the existing methods vs. the proposed.

The fusion network was conditioned by the auxiliary Faster R-CNN detector and is biased towards that specific model. To evaluate whether the network truly generates robust and relevant pedestrian details, we have tested the performance of another pedestrian detector using our fused results (last two columns of Table 1). We applied the state-of-the-art CNN-based object detector YOLO v2 [52], which was never coupled to our fusion network during training.

For evaluation of the methods from literature with respect to pedestrian detection, the fused luminance channel was combined with the chrominance channels of the visible light input, to create a three-color output. The proposed method generates color images and does not require adaptation. In these experiments, we omit the post-processing of the residual output to evaluate the immediate result generated by our approach.

Following the guidelines by the authors of [28], in the last two columns of Table 1 we compare the results using the reasonable subset of the pedestrian samples. Reasonable are all non-occluded pedestrians larger than 55 pixels. We adopt the log-average miss rate (5) as a detection performance measure for consistency with the literature, where lower numbers correspond to better performance. This measure calculates the average proportion of missed pedestrians in the whole set $a_{i}$, for increasing number of false positive outcomes per image.

(5) $$E_{log\_amr}=\exp\left(\frac{1}{n}\sum_{i=1}^{n}\log a_{i}\right)$$

The standard practice adopted in the literature is that the vector of miss rates $a=\{a_{i}\}$ consists of 9 positive values, computed at 9 evenly spaced false positive rates per image (FFPI) in the log-space $\left[10^{-2},10^{0}\right]$.

The proposed method outperforms all the compared methods by a significant margin in terms of its quality as an input for pedestrian detection. In fact, the detection performance using the fused images from the existing methods is even inferior to using only the visible light information, presented in the first row. Moreover, observing that the performance of YOLO on the proposed fusion method is better than that on the compared existing methods, we can conclude that the fusion is indeed generating more informative images. We should note here that in these experiments the detectors were not re-trained to adapt to the fused modalities, and we expect that by re-training the detection performance will improve over the RGB-only detection. However, in this paper we do not focus on the absolute detection performance.

In Table 2 we evaluate cross-dataset and cross-detector performance based on the fusion results generated by our method. As an additional experiment we tested the performance on a second, labeled multi-modal dataset, CVC-14 [29]. We compared the performance of the two pedestrian detectors when they are applied to the original RGB images vs. the fused results of the proposed method.

Compared to KAIST, in CVC-14, the visible light images are grayscale and it has more problems with synchronization and misalignment. However, the number of annotated multi-modal dataset for automotive purposes is very limited and we perform our comparison with the ones that are available. Since this dataset provides separate pedestrian bounding-box annotations for both visible light and thermal images, we first combine them into a single-view bounding boxes by merging the overlapping and keeping the union of the non-overlapping ones. Moreover, we discarded frames with significant synchronization problems when performing our experiments.

The cross-detector and cross-dataset performance results provided in Table 2 indicate that the fused images contain supplemental pedestrian details and they improve the recognition power of both detectors, especially visible in the nighttime examples. Thus, we can conclude that the proposed learning framework is effective for training the network to rely on both visible and thermal inputs. Our fusion network can create artificial details that are consistent to the natural image appearance and boost the visibility of the objects of interest.

Both experiments show that the perceptual quality of the output (measured by the standard metrics) is not a universal indicator of the fusion success, and that the improved low-level image properties do not necessarily improve other higher-level vision tasks performed on the high-quality reconstructed images. Our intended application, using the fused images to warn a human driver of pedestrian presence on the street, is also a high-level recognition task. Focusing on context-based image features in addition to low-level-based ones helps in optimizing the visualization of specific objects of interest. For more realistic method evaluation, quality metrics related to driving applications that closely simulate high-level cognitive perception are needed.

The proposed method was implemented using the MatConvNet toolbox [53], which is a deep learning toolbox developed for MATLAB. We have used an existing MatConvNet implementation of a pre-trained model for Faster R-CNN. To train our fusion network we made the necessary adaptations to connect the two networks as explained above and fix the parameters of Faster R-CNN.

In terms of the processing speed, the trained fusion network fuses a single-precision pair of RGB and LWIR images of size 750 × 600 at about 1.3 frames per second on the Nvidia GeForce GTX 1070 GPU.

## 6. Conclusions and Future Work

In this paper, we have proposed a novel learning-based method for fusion of visible and thermal images with the goal to create intuitive images with improved pedestrian visibility. The proposed method is conceptually different from most recent visible light and thermal image fusion techniques. Our goal was to develop a general image fusion method to enhance object visibility, with the main application in driver-assistance applications for reliable visualization in challenging conditions. The main novelty of this paper is the training framework for the neural network that performs image fusion. The idea is that the training relies both on low-level (similarity) error and a high-level (pedestrian detection ) error to learn to extract relevant features from the inputs and combine them into a more informative output.

The experimental results indicate that using an auxiliary detection performance score as an objective metric can effectively guide the extraction of information from the inputs that is relevant for a specific goal, such as pedestrian visualization in natural images. Moreover, the experiments show that for high-level tasks such as object detection, the optimal input is not necessarily an image which satisfies low-level perceptual metrics. Our approach allows automatic exploration of the best pixel-wise combinations of images from different modalities that contributes towards improved details in pedestrian regions.

As deep learning-based methods are becoming more popular, exhaustively trained models are increasingly more available. Re-using them as auxiliary means to optimize problems from other domains may further extend the applicability of frameworks such as the proposed.

In future work we will experiment with multi-scale generative networks for stronger context modeling. Opportunities for further development lie in the direction of distinguishing between modalities which are suitable for each visibility condition, while still retaining a unified, general, and robust framework. Moreover, we expect that the investigation of solutions that more accurately follow cognitive models of the human visual system will prove beneficial for visualization and improved safety of the ADAS systems. Future work will also involve investigation of the potentials to extend the applicability of the proposed method in different automotive vision tasks, not limited to object detection and visualization.

## Figures and Tables

**Table 1:** Qualitative comparison of the proposed method with ten image fusion methods using samples from the KAIST Pedestrian Benchmark. The metrics are averaged from all day and night images in the test set.

Fusion Method	MI	$Q^{\mathrm{AB}/F}$	IE	AG	SF	Miss Rate (%)	
						Day/Night	
						Faster R-CNN	YOLO v2
No fusion (RGB only)	n/a	n/a	n/a	n/a	n/a	66.28/68.58	57.26/55.95
CNNs IR + VIS [5]	2.00	0.72	**7.47**	6.39	11.16	74.30/74.25	62.58/59.52
Hybrid MSD [6]	1.90	0.69	7.35	**7.10**	**12.3**	74.93/75.59	61.37/60.85
Image Fusion with Guided Filt. [7]	2.32	0.60	7.10	4.36	8.95	85.85/77.71	73.27/59.23
ResNet50 + CA [5]	1.73	0.61	6.81	3.80	7.50	75.36/75.50	64.56/60.11
Image Fusion using Deep Learning [9]	1.93	0.59	6.76	3.73	7.41	75.65/73.74	63.48/60.73
Structure-Aware Image Fusion [10]	3.48	**0.73**	7.45	6.49	11.60	75.75/71.08	64.44/57.82
Image Fusion using CBF Codes [11]	1.92	0.68	7.25	5.85	10.56	78.31/77.23	68.57/63.27
Image Fusion based on FPDE [12]	1.97	0.63	6.51	4.51	8.02	74.05/76.74	59.68/58.19
Two-Scale Image Fusion w/Sal. [13]	1.53	0.68	7.00	6.14	10.73	73.64/70.57	64.03/58.66
MSVD Image Fusion [14]	1.79	0.60	6.77	4.93	8.97	80.01/75.38	65.78/50.49
Proposed	**4.16**	0.70	7.23	5.67	11.19	**52.07/43.25**	**55.85/44.12**

**Table 2:** Cross-detector and cross-dataset performance comparison.

		Log-Average Miss Rate (%)			
		KAIST		CVC-14	
		day	night	day	night
Faster R-CNN	visible	66.28	68.58	69.82	80.26
	fused	52.07	43.25	69.14	63.52
YOLO v2	visible	57.26	55.95	56.81	58.44
	fused	55.85	44.12	60.95	47.19
